# Influence of mean arterial pressure and cardiac output on renal vascular tone reflected by the renal Doppler resistive index in critically ill patients

**DOI:** 10.1186/cc14708

**Published:** 2015-09-28

**Authors:** Raphael Augusto G de Oliveira, Leandro U Taniguchi, Marcelo Park, Pedro V Mendes

**Affiliations:** 1 Hospital das Clínicas, Universidade de São Paulo, SP, Brazil

## Introduction

The renal Doppler resistive index (RI) is a non-invasive tool used to predict acute kidney injury (AKI) and evaluate renal vascular tone in the ICU setting. However, the real impact of hemodynamic parameter variations on RI in critically ill patients is unknown.

## Objective

To evaluate the influence of mean arterial pressure (MAP) and cardiac output (CO) on RI in critically ill patients.

## Methods

Prospective observational study performed in the medical-surgical ICU from August 2014 to December 2014. RI was performed daily until ICU discharge, death or need for renal replacement therapy (RRT). Transthoracic echocardiography was performed immediately after RI analysis to estimate cardiac output using the velocity-time integral (VTI) at the left ventricular outflow tract. All clinical and laboratorial data were obtained routinely during daily ultrasound examinations. Patients with chronic renal disease or on dialysis were excluded. Transient AKI was defined by normalization of renal function within 48 hours of AKI onset. Persistent AKI was defined by nonresolution of AKI within 48 hours of onset or need for RRT.

## Results

**Figure 1:**
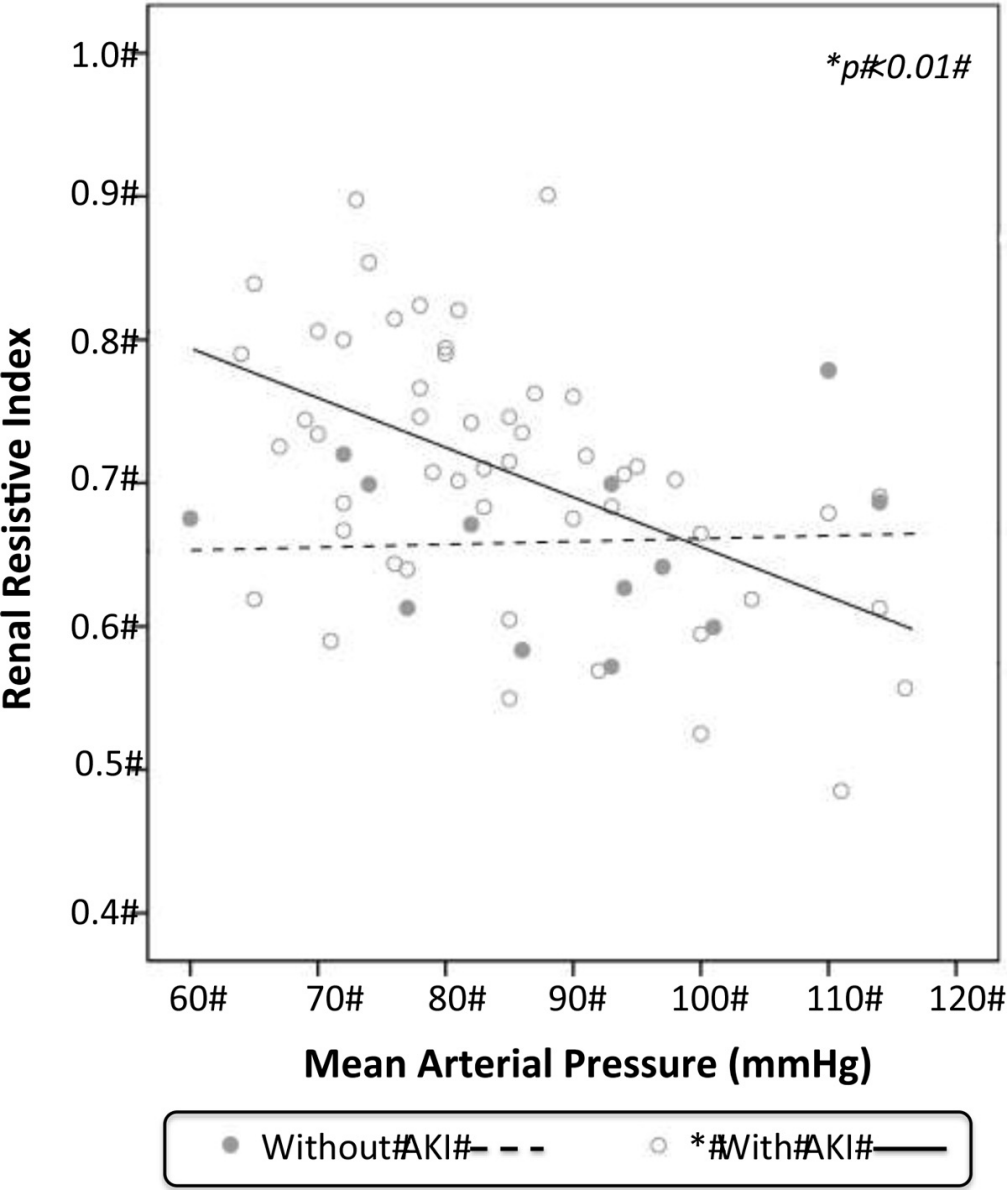
**Relationship between mean arterial pressure and RI**.

Twenty-six patients were included (61 % medical admissions, 77 % male, SAPS 3 of 52 ± 14). Seventy-three percent of patients developed AKI during the ICU stay (35 % had persistent AKI and 15 % required RRT). Patients with persistent AKI had higher values of serum creatinine (1.76 ± 0.82 mg/dl compared with 1.06 ± 0.35 mg/dl in the transient AKI group or 0.79 ± 0.29 mg/dl in the without AKI group, *p *<0.01 between groups) and RI (0.74 ± 0.07 compared with 0.65 ± 0.08 in the transient AKI group or 0.65 ± 0.05 in the without AKI group, *p *<0.01 between groups). There was no difference between mean MAP (88 ± 15 mmHg) in patients without AKI compared with patients with AKI (84 ± 13 mmHg, *p *= 0.429) and between mean CO (7.35 ± 1.53 l/minute) in patients without AKI compared with patients with AKI (7.44 ± 1.70 l/minute, *p *= 0.848). MAP and RI demonstrated a negative correlation (*r *= -0.507, *p *<0.01; Figure 1) in patients with AKI (*r *= -0.455, *p *<0.05 in transient AKI and *r *= -0.372, *p *<0.05 in persistent AKI) and no correlation (*r *= 0.053, *p *= 0.864) in those who did not develop AKI. There was no correlation between CO and RI in patients with AKI (*p *= 0.532) or without AKI (*p *= 0.59).

## Conclusion

We observed a negative correlation between RI and MAP in patients with AKI. No correlation was observed between RI and CO in the different groups.

